# Liraglutide reduces body weight by upregulation of adenylate cyclase 3

**DOI:** 10.1038/nutd.2017.17

**Published:** 2017-05-08

**Authors:** Z Li, Y Liang, N Xia, Y Lai, H Pan, S Zhou, F Jiang, Y He

**Affiliations:** 1 Department of Endocrinology and Metabolism, The First Affiliated Hospital of Guangxi Medical University, Nanning, China; 2 Department of Endocrinology and Metabolism, The Second Affiliated Hospital of Guangxi Medical University, Nanning, China; 3 Department of Hematology, The First Affiliated Hospital of Guangxi Medical University, Nanning, China; 4 Department of Endocrinology and Metabolism, Affiliated Hospital of Guilin Medical College, Guilin, China

## Abstract

**Objective:**

According to recent studies, adenylate cyclase 3 (AC3) is associated with obesity. Liraglutide reduces blood glucose levels and body weight (BW). We performed a 2 × 2 factorial experiment to study the relationships among AC3, liraglutide and obesity and to obtain a more comprehensive understanding of the mechanisms underlying the physiological effects of liraglutide on obesity.

**Methods:**

A high-fat diet was used to induce obesity in C57BL/6J mice. Both the normal and obese mice were treated with liraglutide (1 mg kg^−1^) or saline twice daily for 8 weeks. The hepatic levels of the AC3 and glucagon-like peptide receptor (GLP-1R) mRNAs and proteins were measured by quantitative real-time PCR and western blotting, respectively. The serum AC3 levels were detected using a rat/mouse AC3 enzyme-linked immunosorbent assay kit.

**Results:**

The administration of liraglutide significantly decreased the BW in obese mice and normal control mice. The BW of obese mice exhibited a more obvious decrease. Hepatic AC3 mRNA and protein levels and serum AC3 levels were significantly reduced in obese mice compared with those in normal control mice. The administration of liraglutide significantly increased the hepatic expression of the AC3 and GLP-1R mRNAs and proteins and serum AC3 levels. The hepatic expression of the AC3 mRNA and protein and serum AC3 levels were negatively correlated with BW loss in the liraglutide-treated group. Pearson’s correlation coefficients for these comparisons are *r*=−0.448, *P*=0.048; *r*=−0.478, *P*=0.046; and *r*=−0.909, *P*=0.000, respectively.

**Conclusions:**

Based on our research, liraglutide reduces BW, possibly by increasing the expression of AC3.

## Introduction

Epidemiological studies have estimated that global obesity rates have increased steadily over the past 30 years. In the United States, more than one-sixth of children and adolescents and one-third of adults are considered obese.^1, 2, 3, 4^ Overweight and obesity increase the risk of developing type 2 diabetes (T2D), coronary heart disease, ischaemic stroke and several cancers.^5, 6, 7^ Therefore, pharmacological therapies must be developed to treat obesity.

Based on clinical observations, liraglutide reduces body weight (BW) in patients with T2D.^8^ In addition, liraglutide treatment augments weight loss in overweight and obese older individuals with prediabetes^9^ or without diabetes.^10^ Liraglutide binds to and activates the glucagon-like peptide-1 receptor (GLP-1R) that is the target of endogenous glucagon-like peptide-1 (GLP-1). Proteins in the adenylate cyclase (AC) family catalyse the production of cyclic adenosine monophosphate. The cyclic adenosine monophosphate generated by GLP-1 receptor activation may also directly influence the exocytosis of insulin-containing granules, and this process has been estimated to account for up to 70% of the entire secretory response.^11^ Adenylate cyclase 3 (AC3) is a member of the AC family, and an obesity locus at or near the AC3 gene has been identified in genome-wide association studies.^12, 13, 14, 15^ Additional genetic evidence supports an association between AC3 variants and obesity in both Swedish and Han Chinese populations.^16, 17^ Animal models have also highlighted the importance of AC3 signalling in energy homeostasis, as AC3−/− mice exhibit more fat mass under basal conditions and display a greater susceptibility to obesity induced by consumption of a high-fat diet (HFD).^18^ According to our previous studies, hepatic AC3 expression is upregulated by liraglutide in obese and diabetic mice.^19^

In the present study, we employ a mouse model of obesity induced by feeding HFD to C57BL/6J mice. Furthermore, the effects of twice-daily liraglutide treatments for 8 weeks on hepatic expression of the AC3 and GLP-1R genes and serum AC3 levels, which is easier to assess and detect than hepatic AC3 levels, were also investigated in this mouse model. These results will improve our knowledge of AC3, lead to a more comprehensive understanding of the mechanisms underlying the physiological effects of liraglutide on obesity and provide prospects for new therapeutic interventions for human obesity disorders.

## Materials and methods

### Animal husbandry and analytical procedures

Experimental animals purchased from the Medical Laboratory Animal Centre of Guangzhou Province (Guangzhou, China) were maintained at the Animal Experiment Centre of Guangxi Medical University. The experiments were performed on 4-week-old C57BL/6J mice. The mice were housed in individual cages in a temperature-controlled room on a 12 h light/dark cycle. Mice had free access to standard mouse chow and water. After 1 week, 24 C57BL/6J mice were divided randomly into 2 groups. The normal control group (NC, *n*=12) was fed a normal diet and the HFD group was fed an HFD (34.9% fat and 26.2% protein) for 12 weeks to generate obese mice (O, *n*=12). BW and blood glucose levels were measured weekly. Mice with fasting blood glucose (FBG) levels >13.9 mmol l^−1^ (250 mg dl^−1^) for three consecutive days were considered diabetic.^20^ Mice with BW that exceeded the normal weight by at least 20% were considered obese. After the mouse model of obesity was successfully established, the mice were all fed a normal diet and were subdivided into the following groups: NC+saline (NC+S), NC+liraglutide (NC+L),O+saline (O+S) and O+liraglutide (O+L). The NC+L and O+L groups were subcutaneously injected with the GLP-1 analogue liraglutide at a dosage of 0.1 mg kg^−1^ per 12 h. The NC+S and O+S groups were treated with subcutaneous injections of the same dosage of saline. After 8 weeks, the mice were fasted overnight and anaesthetized with sodium pentobarbital (50 mg kg^−1^, intraperitoneal). Blood was obtained via the angular vein. Plasma was separated by centrifugation at 4 °C and stored at −20 °C until further analysis. The livers were immediately dissected, frozen in liquid nitrogen and stored at −80 °C until further analysis. All of the animal experiments and care procedures were conducted under the Guidelines of the Animal Ethics Committee of Guangxi Medical University.

### Serum insulin, serum AC3 and blood glucose levels

Mouse insulin levels were measured with a rat/mouse insulin enzyme-linked immunosorbent assay (ELISA) kit (Shanghai JiNing Industrial Co., Ltd, Shanghai, China). Serum AC3 levels were measured with a rat/mouse AC3 ELISA kit (TSZ Biological Trade Co., Ltd, San Francisco, CA, USA). Blood glucose levels were detected using a glucose metre (Johnson & Johnson, New Brunswick, NJ, USA). The formula (fasting insulin (mU l^−1^) × fasting glucose (mmol l^−1^))/22.5 was used to calculate homeostasis model assessment of insulin resistance (HOMA-IR) scores.^21^

### Reverse transcriptase-PCR and real-time reverse transcriptase-PCR

The ID number of the mouse adcy3 gene in mouse is 104111, and the adcy3 mRNA sequence is NM_138305.3. The ID number of the mouse GLP-1R gene is 14652, and the GLP-1R mRNA sequence is NM_021332.2. Total RNA was isolated from the liver and complementary DNAs were synthesized using a Thermo Reverse Transcriptase Kit (Thermo Scientific, Waltham, MA, USA). PCR was performed in a Light Cycler (Applied Biosystems, Foster City, CA, USA) at 95 °C for 10 min followed by 40 cycles of 95 °C for 15 s and 60 °C for 45 s. The sequences of the sense and antisense primers for AC3 are 5′-GGACACGCTCACAAACATC-3′ and 5′-GCCACATTGACCGTATTGC-3′, respectively. The sequences of the sense and antisense primers used to amplify GLP-1R are 5′-ATGGTGGCTATCCTGTACTGCTTTG-3′ and 5′-GCTGCTGGTGGGACACTTGA-3′, respectively. The glyceraldehyde 3-phosphatedehydrogenase (gapdh) mRNA levels, which were analysed as an internal control, were obtained using the following primers: 5′-TGTGTCCGTCGTGGATCTGA-3′ (sense) and 5′-TTGCTGTTGAAGTCGCAGGAG-3′ (antisense). The relative copy number was calculated using the threshold crossing point (Ct) calculated by the LightCycler software combined with the ΔΔCt calculations.

### Western blotting

Western blot analyses were performed as previously described.^22^ Isolated liver tissues were homogenized on ice in homogenization buffer. Protein concentrations were measured using a bicinchoninic (BCA) assay kit (Thermo Scientific) according to the manufacturer’s instructions. Samples (40–60 mg) were boiled at 95 °C for >5 min and then placed on ice and loaded onto 7.5% polyacrylamide gels. The gels were transferred to a nitrocellulose membrane (Millipore, Billerica, MA, USA) and blocked with 5% milk in phosphate-buffered saline with 0.05% Tween-20 for 1 to 2 h. Blots were incubated with a polyclonal AC3 antibody (1:1000; Sigma-Aldrich Co., LLC, Shanghai, China) and polyclonal GLP-1R antibody (1:1000; Bioworld Technology Co., Ltd, Nanjing, China) overnight at 4 °C and then with a horseradish peroxidase-conjugated goat anti-rabbit lgG for 1 h at room temperature. The blots were developed with an enhanced chemiluminescence reagent detection kit (Millipore) according to the manufacturer’s instructions.

### Statistical analysis

Quantitative data are presented as mean±s.d. Significant differences were analysed using Student’s *t*-test, one-way analysis of variance, a factorial analysis or Pearson’s correlation analysis where appropriate. A homogeneity of variance test was applied in all cases analysed with Student’s *t*-test, one-way analysis of variance or a factorial analysis. The *P*-values of <0.05 were considered statistically significant. All analyses were performed using SPSS 17.0 (SPSS Inc., Chicago, MI, USA).

## Results

### Effects of liraglutide on the general condition and metabolism of the mice

**Figure 1:**
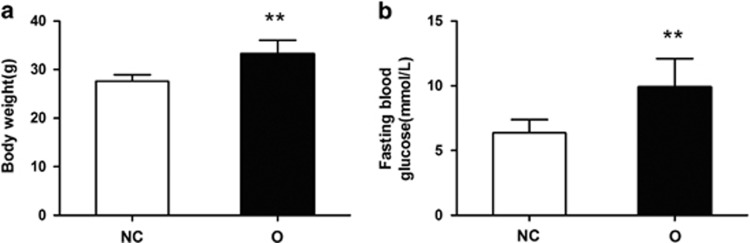
BWs and FBG levels in mice before liraglutide treatment. The BWs and FBG levels in the normal control group and obese group are shown in (**a**) and (**b**), respectively. Values are presented as mean±s.d. (*n*=12 per group). **P<0.01 compared with the control group.

**Figure 2:**
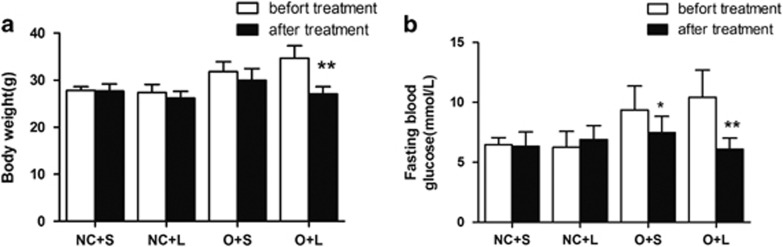
BWs and FBG levels in mice before and after liraglutide or saline treatment. The BWs and FBG levels observed before and after liraglutide or saline treatment are summarized in (**a**) and (**b**), respectively. Values are presented as means±s.d. (*n*=6 per group). *P<0.05; **P<0.01.

**Figure 3:**
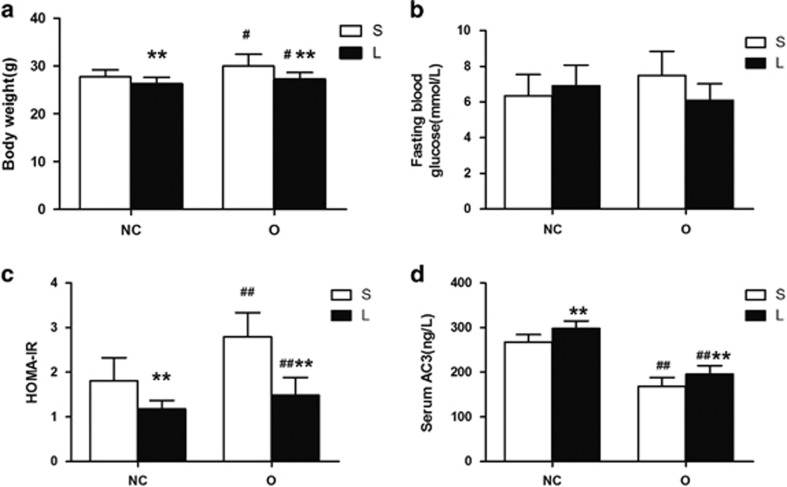
BWs, FBG levels, HOMA-IR scores and serum AC3 levels in mice treated with liraglutide (L) or saline (S). The BWs, FBG levels, HOMA-IR scores and serum AC3 levels observed after treatment with liraglutide or saline are summarized in (**a–d**), respectively. The *P*-values for the effect of the interaction between obesity and liraglutide treatment on BWs, FBG levels, HOMA-IR scores and serum AC3 levels are 0.350, 0.065, 0.008 and 0.736, respectively. Values are presented as means±s.d. (*n*=6 per group). *P<0.05, **P<0.01 for the comparison of the saline and liraglutide groups; ^#^*P*<0.05, ^##^*P*<0.01 for the comparison of the normal and obese groups.

We examined the BW, FBG and HOMA-IR to evaluate the animals’ metabolic responses to liraglutide. Before treatment, the mice in the NC group exhibited significantly reduced BWs compared with the mice in the O group (Figure 1a). The FBG levels were elevated in the O group compared with the NC group (Figure 1b). The BWs in the O+L group and FBG levels in the O+S and O+L groups were significantly decreased after treatment (Figure 2). The BW and FBG of the O+L group were reduced by an average of 21.8% and 45.9%, respectively, compared with the BW and FBG in the O+S group that were reduced by an average of 5.8% and 20.2%, respectively. The factorial analysis revealed significant differences in the BWs between the normal control mice and the obese mice and between the liraglutide-treated mice and the saline-treated mice (Figure 3a). Significant differences in the FBG were not observed between the normal control mice and the obese mice or between the liraglutide-treated mice and the saline-treated mice (Figure 3b). The HOMA-IR scores were significantly different between the normal control mice and the obese mice and between the liraglutide-treated mice and the saline-treated mice (Figure 3c). The calculated HOMA scores improved by an average of 47.1% in the O+L group compared with an average of 34.8% in the NC group.

### Effects of liraglutide on the serum AC3 levels in mice

We evaluated the serum AC3 levels that were easier to assess and detect than the hepatic AC3 levels. The serum AC3 levels were significantly reduced in the obese mice compared with the normal control mice. The liraglutide treatment significantly increased the serum AC3 levels in the obese mice and the normal control mice. In particular, the serum AC3 levels were increased by an average of 16.3% in the O+L group and by 11.6% in the normal control mice (Figure 3d). The serum AC3 levels were negatively correlated with BW (*r*=−0.464, *P*=0.022) and the HOMA-IR score (*r*=−0.512, *P*=0.011). Moreover, the serum AC3 levels were negatively correlated with BW loss in the saline group (*r*=−0.558, *P*=0.060) and liraglutide group (*r*=−0.909, *P*=0.000).

### Effects of liraglutide on the hepatic levels of the AC3 and GLP-1R mRNAs and proteins in mice

**Figure 4:**
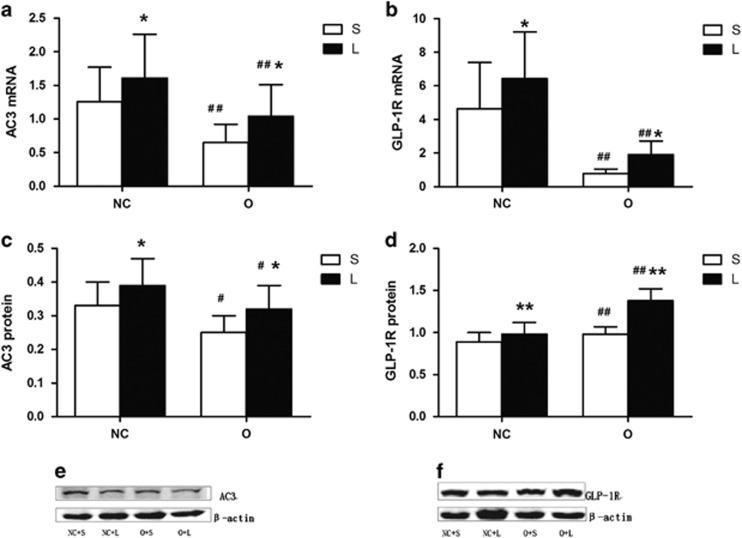
Expression of the AC3 and GLP-1R mRNAs and proteins in the liver after treatment with liraglutide (L) or saline (S). Expression of the AC3 and GLP-1R mRNAs and proteins in liver tissues of mice treated with liraglutide or saline are shown in (**a–d**), respectively. The image of AC3 and GLP-1R from western blotting experiments is represented in (**e** and **f**). The *P*-values for the effect of the interaction between obesity and liraglutide treatment on the hepatic AC3 mRNA, GLP-1R mRNA, AC3 protein and GLP-1R protein levels are 0.724, 0.96, 0.846 and 0.005, respectively. Values are presented as means±s.d. (*n*=8 per group, when detecting the expression levels of the AC3 and GLP-1R mRNAs; *n*=6 per group, when detecting the expression levels of the AC3 and GLP-1R proteins). *P<0.05, **P<0.01 for the comparison between the saline and liraglutide groups; ^#^*P*<0.05, ^##^*P*<0.01 for the comparison between the normal and obese groups.

We examined the levels of the AC3 and GLP-1Rs mRNA and proteins in the liver tissue (Figures 4a–f). The levels of the AC3 and GLP-1R mRNAs and proteins were significantly reduced in the obese mice compared with the normal control mice. The liraglutide treatment significantly increased the AC3 and GLP-1R mRNA and protein levels. The levels of the AC3 mRNA were positively correlated with the levels of the GLP-1R mRNA (*r*=0.558, *P*=0.005). Furthermore, the levels of the AC3 mRNA were negatively correlated with BW (*r*=−0.557, *P*=0.005) and the HOMA-IR score (*r*=−0.540, *P*=0.006), and the AC3 mRNA levels were negatively correlated with BW loss in the saline group (*r*=−0.083, *P*=0.761) and liraglutide group (*r*=−0.448, *P*=0.048). The levels of the AC3 protein were negatively correlated with the BW (*r*=−0.524, *P*=0.009) and the HOMA-IR score (*r*=−0.635, *P*=0.001), and the levels of the AC3 protein were negatively correlated with BW loss in the saline group (*r*=−0.105, *P*=0.746) and liraglutide group (*r*=−0.478, *P*=0.046). The levels of the AC3 mRNA and protein were negatively correlated with the BW and the HOMA-IR score, consistent with the results of our previous study.^19^ The serum AC3 levels were positively correlated with the AC3 protein levels (*r*=0.567, *P*=0.004).

## Discussion

In this study, obesity was successfully induced in mice using HFD. The FBG of these obese mice was increased compared with the NC group. After obesity was successfully induced in the mice, all of the mice were fed a normal diet. The BW and FBG levels were significantly decreased in the obese mice treated with liraglutide or saline, and a factorial analysis did not reveal significant differences in the FBG between the mice treated with liraglutide or saline. This finding is explained by the improved BW and blood glucose levels in mice fed a normal diet and the lack of an effect of liraglutide on normal blood glucose levels. In addition, the liraglutide treatment decreased the BW and HOMA-IR scores in obese mice and normal control mice. The BW and HOMA-IR scores exhibited a greater decrease in obese mice.

We explored the impact of the GLP-1 analogue liraglutide on the levels of the GLP-1R and AC3 proteins and mRNAs in the liver and serum from the obese mice. GLP-1R is the target for endogenous GLP-1. The liraglutide treatment increased the levels of the GLP-1R and AC3 mRNAs and proteins, and the levels of the GLP-1R and AC3 mRNAs were positively correlated. Thus, the expression of the AC3 gene is induced by liraglutide to some extent. A genetic association study of the AC3 gene in Swedish patients with T2D and obese subjects found that polymorphisms in the AC3 gene protect against obesity but are not associated with plasma glucose and insulin levels in patients with T2D.^16^ In our present study, liraglutide did not affect the FBG of the obese mice, and AC3 expression was not associated with the FBG and insulin levels. Liraglutide improves the blood glucose levels and reduces BW potentially via different mechanisms.

GK rats, a hereditary nonobese animal model of T2D, exhibit elevated AC3 expression levels in the pancreatic islets and significantly reduced BWs compared with Wistar rats.^23^ Mice carrying the AC3Jll mutant allele exhibit increased AC3 activity in response to forskolin and are protected from HFD-induced obesity.^24^ In the present study, liraglutide upregulated the hepatic and serum AC3 levels. Moreover, the hepatic and serum AC3 levels were negatively correlated with the BW and HOMA-IR score. Moreover, the regression analysis identified different effects of AC3 changes against BW loss on the saline and liraglutide groups and with a stronger relationship for the latter. Thus, we believe that liraglutide reduces BW by increasing the expression of AC3.

Based on human genetics and animal models, AC3 may play an important role in energy homeostasis. Data from our mouse model provide evidence that supports this hypothesis. However, the underlying mechanisms remain unclear. Thus, additional studies are required to provide new insights into this subject.

## Conclusions

In this study, the AC3 levels were significantly reduced in obese mice compared with normal control mice. The hepatic and serum AC3 levels were significantly increased in response to the liraglutide treatment and the hepatic AC3 mRNA and protein levels were negatively correlated with BW and the HOMA-IR score. Moreover, the AC3 levels were negatively correlated with BW loss in the liraglutide-treated group. Thus, we conclude that liraglutide reduces BW by increasing the expression of AC3.
